# Mitochondria-Targeted Hydrogen Sulphide Delivery via an Adhesive Hydrogel Modulates Inflammation and Oxidative Stress in Diabetic Wounds

**DOI:** 10.3390/gels12030251

**Published:** 2026-03-17

**Authors:** Mandeep Kaur Marwah, Hala Shokr, Yukta Sameer Hindalekar, Mohamad Anas Al Tahan, Karan Rana, Lissette Sanchez-Aranguren, Maymunah Sarr, Jacob Baxandall, Katy Mcgonigal, Bahareh Hassanzadeh, Shakil Ahmad, Sami A. Al-Ani, Jeevan Singh Lall, Harmony C. K. Cheema, Kavun Dhesi, Keqing Wang, Irundika H. K. Dias, Srikanth Bellary, Anisa Mahomed

**Affiliations:** 1 Aston Medical School, College of Health and Life Sciences, Aston University, Birmingham B4 7ET, UK; 2 Aston Research Centre for Health in Ageing, Aston University, Birmingham B4 7ET, UK; 3 Pharmacy Division, School of Health Sciences, Faculty of Biology, Medicine and Health, The University of Manchester, Manchester M13 9PR, UK; 4 School of Biosciences, College of Health and Life Sciences, Aston University, Birmingham B4 7ET, UK; 5 Chemical Engineering and Biotechnologies, College of Engineering and Physical Sciences, Aston University, Aston Triangle, Birmingham B4 7ET, UK; 6 The Jersey Practice, Heston Health Centre, Hounslow TW5 9ER, UK

**Keywords:** AP39 biological activity, controlled release, inflammation, diabetic wound healing, hydrogel, topical drug delivery

## Abstract

Chronic diabetic wounds are challenging to treat due to persistent inflammation, oxidative stress, impaired angiogenesis, and dysregulated matrix remodelling. Hydrogen sulphide (H_2_S) has emerged as a therapeutic mediator with antioxidant, anti-inflammatory, and pro-angiogenic properties; however, its clinical translation is limited by volatility and a short biological half-life. Controlled delivery systems, such as hydrogels, are therefore required to harness its potential. This study aimed to develop and evaluate a sodium 2-acrylamido-2-methylpropane sulfonate (Na-AMPS)-based adhesive hydrogel incorporating AP39, a mitochondria-targeted H_2_S donor, for sustained localised delivery and promotion of wound healing. Hydrogel formulations were characterised for rheological behaviour, adhesion, swelling, and AP39 release. Cytocompatibility was assessed in human umbilical vein endothelial cells (HUVECs); human dermal fibroblasts, adult (HDFa); and keratinocytes. Anti-inflammatory, antioxidant, and matrix-modulatory effects were evaluated via interleukin-6 and 8 (IL-6/IL-8) secretion, reactive oxygen species (ROS) levels, mitochondrial membrane potential, matrix metalloproteinase-9 (MMP-9), and transforming growth factor-beta (TGF-β). Functional wound healing activity was assessed using tube formation and scratch assays in endothelial cells. AP39-loaded hydrogels exhibited predominantly elastic, shear-thinning behaviour, strong adhesion, rapid hydration, and sustained release of AP39 (11.63 ± 1.20% over 24 h). Across all cell types, 500 nM concentrations of AP39 were well tolerated. In diabetic-like stress conditions, AP39 significantly decreased ROS in HUVECs (50122 ± 5999 to 33,087 ± 1865 AU; *p* < 0.0001) and HDFa cells (41,367 ± 4225 to 29,813 ± 2406 AU; *p* < 0.0001). AP39 improved mitochondrial membrane potential in both cell types (*p* < 0.01–0.001) and decreased pro-inflammatory cytokines. IL-6 decreased in HUVECs (96.05 ± 4.22 pg/mL to 60.99 ± 4.21 pg/mL; *p* < 0.0001) and HDFa cells (77.54 ± 8.94 pg/mL to 52.25 ± 6.78 pg/mL; *p* < 0.001), whilst in HDFa cells, MMP-9 was reduced (419.4 ± 25.51 pg/mL to 174 ± 15.1 pg/mL; *p* < 0.0001). Finally, wound closure was enhanced in HUVECs. The AP39-loaded Na-AMPS hydrogel represents a multifunctional wound dressing capable of controlled H_2_S delivery, mechanical stability, and biological activity to support tissue repair in diabetic wound environments. These results highlight this gel’s therapeutic potential for diabetic wound treatment.

## 1. Introduction

Diabetic foot ulcers (DFU) are a major global health concern, placing substantial financial strain on healthcare systems. These wounds frequently develop as a consequence of long-term complications associated with diabetes, including persistent inflammation [1], elevated levels of reactive oxygen species (ROS) [2] and compromised vascular function [3]. Such pathological factors collectively impair healing, promote tissue degradation, and increase susceptibility to infection, often resulting in extended hospital stays and a higher risk of lower-limb amputation. The economic burden is significant, with costs in the United States approaching $13 billion annually [4]. Among diabetic patients, the annual incidence of foot ulcers is estimated at 2.5–5%, while the lifetime risk ranges from 15 to 20% [5,6]. Despite their prevalence and impact, current therapeutic strategies largely focus on symptomatic management rather than addressing the underlying mechanisms driving poor wound repair [7]. This underscores the urgent requirement for therapies that simultaneously address inflammation, oxidative imbalance, and impaired tissue remodelling to improve healing in DFU.

Hydrogen sulphide (H_2_S) is a potent endogenous signalling molecule with antioxidant, anti-inflammatory, and pro-angiogenic properties, all of which are essential for effective wound repair [8,9,10]. Exogenous administration of H_2_S can supplement deficient or locally insufficient levels in chronic wounds. However, the clinical application of free H_2_S is limited by volatility and a very short half-life, which restrict sustained activity at the wound site [11]. AP39, a mitochondria-targeted H_2_S donor, overcomes these challenges by delivering H_2_S directly to the mitochondria, the primary site of ROS generation and energy production [12]. Targeting H_2_S delivery specifically to mitochondria is particularly critical in the context of diabetic wounds, where sustained hyperglycaemia and sustained inflammation profoundly disrupt mitochondrial homeostasis. In diabetes, mitochondrial dysfunction is characterised by impaired electron transport chain activity, loss of membrane potential, reduced ATP generation, and excessive production of mitochondrial ROS (mtROS). These abnormalities drive oxidative damage, amplify pro-inflammatory signalling, and promote apoptosis, collectively contributing to endothelial dysfunction, impaired fibroblast activity, delayed keratinocyte migration, and ultimately defective wound healing [13]. Mitochondria-targeted H_2_S donors such as AP39 are uniquely positioned to address these defects by restoring mitochondrial bioenergetics and redox balance at their source [12]. In parallel, H_2_S enhances the activity of mitochondrial antioxidant systems, limits mtROS overproduction, and preserves mitochondrial membrane integrity, suppressing the release of apoptosis-inducing factors such as cytochrome c [14]. Further, mitochondrial H_2_S signalling exerts important anti-inflammatory and cytoprotective actions. By modulating redox-sensitive pathways, including Nuclear Factor kappa-light-chain-enhancer of activated B cells (NF-κB) and NOD-like receptor family pyrin domain-containing 3 (NLRP3) inflammasome activation, AP39 attenuates chronic inflammatory signalling that is characteristic of diabetic wounds [15,16,17]. Additionally, H_2_S promotes mitochondrial biogenesis and mitophagy, supporting the removal of damaged mitochondria and maintaining a healthy mitochondrial network essential for cellular repair processes.

Despite its therapeutic potential, AP39 is chemically unstable and diffuses rapidly from the administration site, limiting sustained local H_2_S levels [18]. Delivering AP39 effectively therefore requires strategies that stabilise the compound and provide controlled, prolonged release, ideally mimicking the slow, regulated signalling of endogenous H_2_S [19]. Achieving sustained, localised delivery is particularly challenging in chronic wounds, where poor drug solubility, rapid degradation, and limited retention often reduce therapeutic efficacy. Several drug delivery strategies have been explored to address these limitations, including liposomes, polymeric nanoparticles, nanoemulsions, polymeric films, and conventional ointments or creams [11]. While these systems can enhance drug stability or penetration, they often present limitations such as limited drug loading capacity, rapid drug diffusion away from the wound site, mechanical instability, or poor ability to maintain an optimal moist wound environment [20,21]. Hydrogels have emerged as promising alternatives for dermal drug delivery due to their three-dimensional polymeric network structure and high water content, which allow them to mimic the extracellular matrix and provide a hydrated environment conducive to wound healing [11,21].

Among the various hydrogel systems investigated, Na-AMPS-based hydrogels, formed by radical polymerisation of 2-acrylamido-2-methylpropane sulfonic acid or its sodium salt in aqueous media, have gained interest for biomedical and dermal applications [22]. The sulfonate groups in Na-AMPS confer negative charges that can stabilise drug carriers such as nanoparticles or micelles [23,24], while the hydrophilic backbone promotes skin hydration, softening the stratum corneum and enhancing transdermal penetration [25,26]. These gels are mechanically robust, cytocompatible, and chemically stable, and their network architecture can be tuned to achieve controlled release of incorporated therapeutics without compromising elasticity or comfort [27]. Additionally, their swelling properties allow efficient absorption of wound exudate and maintain a moist microenvironment that facilitates tissue repair [28]. This combination of features makes Na-AMPS hydrogels particularly suitable for delivering hydrophobic, labile compounds such as AP39. By embedding drug carriers within the hydrogel network, solubility limitations can be overcome, while sustained, localised release supports prolonged therapeutic activity at the wound site.

In this work, we engineered a Na-AMPS hydrogel loaded with AP39 to provide controlled, mitochondria-targeted H_2_S delivery while maintaining hydrogel stability and bioadhesive properties. To the best of our knowledge, this is the first study to explore the incorporation of the mitochondria-targeted H_2_S donor AP39 within a Na-AMPS hydrogel platform for topical delivery in the context of diabetic wound healing. The formulation was characterised for release kinetics, mechanical and adhesive performance, and cytocompatibility. Functional assays in HUVECs and HDFa cells under hyperglycaemic and inflammatory conditions were used to assess the impact of AP39 on oxidative stress, inflammatory signalling, and extracellular matrix remodelling. This study aims to provide proof-of-concept that Na-AMPS hydrogels can serve as a multifunctional platform for enhancing local H_2_S bioavailability and supporting wound healing processes in diabetic foot ulcers.

## 2. Method

### 2.1. Materials

1-Hydroxycyclohexyl phenyl ketone (Omnirad 184, catalogue #405612), sodium 2-acrylamido-2-methylpropane sulfonate (Na-AMPS, catalogue #655821) and propylene glycol (catalogue #W294004) were acquired from Sigma-Aldrich (Gillingham, UK). AP39 was obtained from Cayman Chemical (catalogue #17100, CAS #1429173-57-8; Ann Arbor, MI, USA). Polyethylene glycol 400 (PEG-400) diacrylate (SR344) was acquired from Arkema (Colombes, France). Poloxomer 184 (Pluracare^®^ L64G) was obtained from BASF (Ludwigshafen, Germany). Tumour necrosis factor-alpha (TNF-α; catalogue #210-TA-005) was obtained from R&D Systems (Abingdon, UK). Ultrapure water was prepared using a Milli-Q purification system (Millipore, Billerica, MA, USA). Other chemicals, including ethanol, isopropanol, and phosphate-buffered saline, were purchased from Fisher Scientific, Loughborough, UK.

### 2.2. Hydrogel Preparation

The adhesive hydrogel was prepared as described previously [29]. The hydrogel formulation used in this study was iteratively adjusted to accommodate drug incorporation while ensuring that the final hydrogel remained adhesive to the substrate and visibly cohesive during handling and application. Briefly, the formulation comprised sodium 2-acrylamido-2-methylpropane sulfonate (Na-AMPS), which provided intrinsic hydrophilicity, comprising 75.68% (*w*/*w*) of the formulation. Propylene glycol was included at 5.1% (*w*/*w*) to enhance flexibility and improve water retention. To facilitate incorporation of the hydrophobic compound AP39 (0.02% *w*/*w*), Poloxamer 184, a non-ionic surfactant, was included at 18.1% (*w*/*w*). Photopolymerisation was commenced by combining Omnirad 184 as the photoinitiator with polyethylene glycol diacrylate (PEG-400) as the crosslinker, together comprising 1.1% (*w*/*w*) of the formulation. The mixture was thoroughly homogenised under protection from ambient light by aluminium foil. Prior to curing, any entrapped air was removed by degassing under a nitrogen stream for 2 min. The solution was then cast onto a silicone substrate and exposed to ultraviolet (UV) light, resulting in the formation of a lightly cross-linked adhesive hydrogel.

### 2.3. Hydrogel Characterisation

#### 2.3.1. Oscillatory Rheological Analysis

Hydrogel viscoelastic behaviour was characterised using a dynamic shear rheometer (Kinexus, Malvern Instruments Malvern, UK) operated in oscillatory mode. Key rheological parameters, including the storage modulus (G′), loss modulus (G″), and complex viscosity (η*), were determined. Measurements were performed within the linear viscoelastic region at a fixed strain amplitude of 2%. Frequency sweep experiments were carried out over a range of 5–30 Hz using a parallel plate geometry (20 mm diameter) at 36 °C. The measurement gap was adjusted according to sample thickness while maintaining comparable normal force across all samples.

#### 2.3.2. Evaluation of Hydrogel Adhesive Performance

Gel adhesion was quantified using a mechanical testing system (Instron, Norwood, MA, USA) configured for a ball tack adhesion test and fitted with a load cell of 50 N. Circular gel specimens (2.5 cm diameter) were prepared and evaluated for their detachment force from a rigid substrate to mimic practical application and removal conditions. A 1-inch stainless steel spherical probe was lowered onto the hydrogel surface to establish contact (compression phase), followed by probe withdrawal until complete separation (tensile phase). Tests were performed at displacement rates of −0.50 mm/s during compression and 0.50 mm/s during retraction, with a 2.5 g trigger force used to prompt data acquisition. The peak force recorded during detachment was taken as a measure of adhesive strength, important in understanding if the balance between secure tissue adhesion and atraumatic removal required for wound dressing applications has been achieved.

#### 2.3.3. Swelling Characteristics of Hydrogel Formulations

Hydrogel formulations were evaluated for their swelling behaviour by submerging 1 g of each sample in water at ambient temperature. Measurements were taken at predetermined time points between 0 and 40 min. Based on previous work with similar Na-AMPS hydrogels, equilibrium swelling was reached within 30 min, after which no significant increase in mass was observed [29]. Samples were withdrawn, and surface moisture was removed by blotting with filter paper and weighed to obtain the increased mass. The swelling ratio (%) was calculated with the following equation:

$$\%\;Swelling\;=\;\frac{W_{f}-W_{i}}{W_{i}}\;\times 100$$

*W_i_* is the hydrogel weight prior to submerging in water, and *W_f_* is the hydrogel weight at the specified time point.

#### 2.3.4. Long-Term Stability of AP39 in Hydrogel Determination 

**Figure 1:**
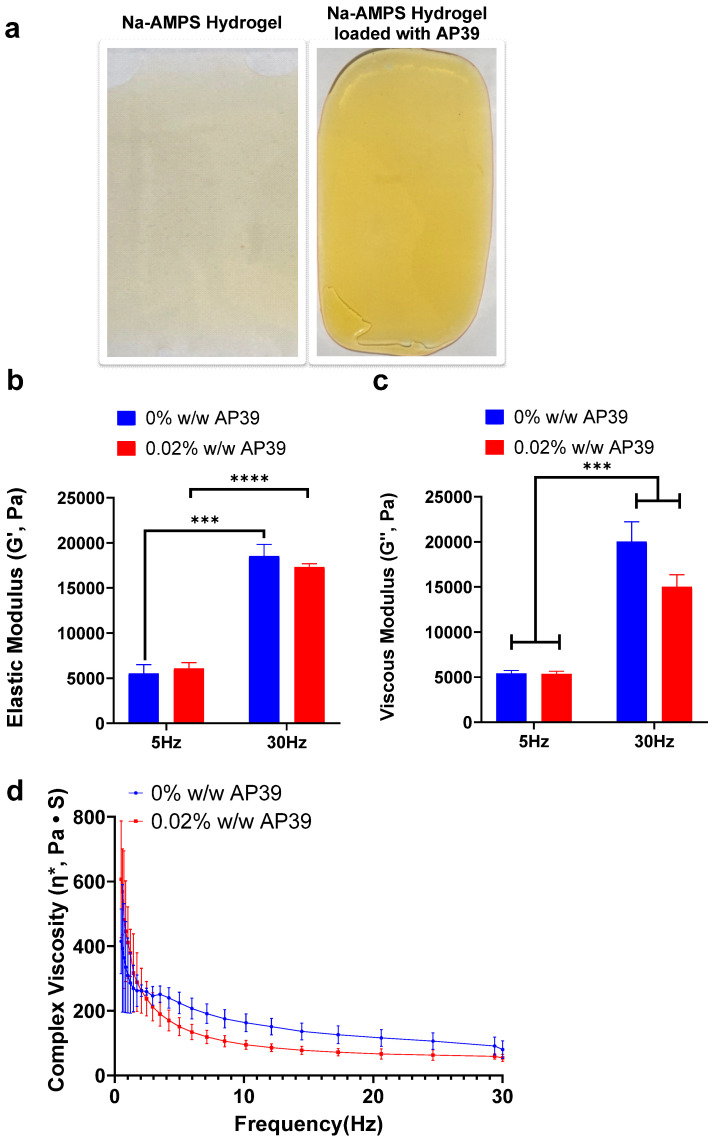
Rheological characterisation of AP39-loaded Na-AMPS hydrogels confirms viscoelastic and shear-thinning properties suitable for topical application. (**a**) Representative images of Na-AMPS-based hydrogel formulations—(i) blank hydrogel (without AP39) and (ii) hydrogel containing 0.02% (*w*/*w*) AP39—both displaying uniform appearance and visual homogeneity. Hydrogels were prepared using Na-AMPS (75.68% *w*/*w*), propylene glycol (5.1% *w*/*w*), and Poloxamer 184 (18.1% *w*/*w*). (**b**) Elastic modulus (G′) and (**c**) viscous modulus (G″) of hydrogels measured at 5 Hz and 30 Hz show a frequency-dependent increase, consistent with typical viscoelastic materials. (**d**) Complex viscosity (η*) profiles indicate shear-thinning behaviour of AP39-loaded hydrogels, which facilitates spreadability under stress. Hydrogels were prepared using Na-AMPS (75.68% *w*/*w*), propylene glycol (5.1% *w*/*w*), Poloxamer 184 (18.1% *w*/*w*), and AP39 (0.02% *w*/*w*). Data represent mean ± SD (*n* = 4). Comparisons were performed using unpaired Student’s *t*-tests. Statistical significance: *** *p* < 0.001, **** *p* < 0.0001.

Hydrogels were prepared as described in Section 2.2, and visual inspection confirmed homogeneity and consistent appearance across all formulations, with no visible phase separation, particulate matter, or colour change (Figure 1a). The stability of AP39 within the hydrogel formulation was assessed by quantifying drug content as a percentage of the theoretical loading over an 84-day period. Samples were stored in a stability chamber (Firlabo, Meyzieu, France) maintained at 25 °C and 60% relative humidity. At each time point, gel samples were trimmed to size and weighed (≤100 mg), then placed in acetonitrile with ceramic beads. Samples were homogenised with a VelociRuptor Microtube Homogeniser (V2) at ambient temperature to ensure complete disruption of the matrix and full drug release. The homogenates were then centrifuged (16,000 RCF), and AP39 in the supernatant was quantified by HPLC-UV as previously described [18].

#### 2.3.5. In Vitro Release of AP39 from Hydrogel Preparations

To determine the *in vitro* release kinetics of AP39 from the gels, a permeable insert model was used, as previously described [29]. This model allowed comparison of AP39 release from both the AP39 solution and the hydrogel. A cell culture insert (Thincert^TM^, cylindrical, 4 cm^2^, 400 µm pore size, BD Biosciences, Woburn, MA, USA) was prepared with 1 mL of the formulation and inserted into the wells of a 6-well Thincert^TM^ plate, each of which were primed with 4 mL of phosphate-buffered saline (PBS) and ethanol mix (90:10) as the release medium. Plates were maintained at 35 °C on a shaking platform to simulate physiological diffusion conditions. At predetermined intervals over 6 h, 0.5 mL aliquots were collected and immediately replaced with fresh PBS/ethanol to maintain sink conditions. AP39 release was quantified using HPLC-UV as described previously [30]. Briefly, quantification was carried out using a Shimadzu LC-2030C Plus system equipped with a Phenomenex HyperClone^TM^ C18 column, Phenomenex, Torrance, CA, USA. An isocratic method was used with the mobile phase consisting of an 87.5:12.5 ratio of 0.01% TFA in acetonitrile to 0.01% TFA in water delivered at a flow rate of 1.25 mL/min.

### 2.4. Cell Culture

Human umbilical vein endothelial cells (HUVECs) (PromoCell, Cat. #C-12203) were grown in Endothelial Growth Medium-2 (EGM-2; PromoCell, Cat. #C-22211), complemented with a growth factor combination including epidermal growth factor (5 ng/mL), foetal calf serum (0.02 mg/mL), basic fibroblast growth factor (10 ng/mL), insulin-like growth factor (20 ng/mL), vascular endothelial growth factor (0.5 ng/mL), heparin (22.5 μg/mL), ascorbic acid (1 μg/mL), and hydrocortisone (0.2 μg/mL) (Supplement Kit, PromoCell, Cat. #C-39211) [29]. In addition, penicillin/streptomycin (5 mL, Lonza, Cat. #LZDE17-602E) was also included. Cells were incubated at 37 °C with 5% CO_2_, with medium changes every 48–72 h, and experiments were conducted using cells at passages ≤5.

Human Dermal Fibroblasts (HDFa; Thermo Fisher, Waltham, MA, USA, Cat. #C0135C) were grown in Human Fibroblast Expansion Basal Medium complemented with Low Serum Growth Supplement, 2% (*v*/*v*) foetal bovine serum, basic fibroblast growth factor (3 ng/mL), hydrocortisone (1 μg/mL), human epidermal growth factor (10 ng/mL), and heparin (10 μg/mL) (Thermo Fisher, Cat. #M106500 and S00310, respectively). Cells were similarly maintained at 37 °C in a humidified environment with 5% CO_2_ and experiments were conducted using cells at passages ≤15.

HaCaT human keratinocytes (Cytion, 300493) were maintained in Dulbecco’s Modified Eagle Medium (DMEM) supplemented with 4.5 g/L glucose, 3.7 g/L sodium bicarbonate (NaHCO_3_) and 4 mM L-glutamine and cultured at 37 °C in a humidified environment containing 5% CO_2_.

In subsequent cell-based assays, blank hydrogels were not included; however, previous studies using similar Na-AMPS formulations demonstrated that the hydrogel matrix alone does not affect any of the outcomes measured here, and therefore the untreated control served as a true control.

#### 2.4.1. Cell Viability of HUVEC, HDFa and HaCaT Following AP39 Treatment

AP39 is a reducing agent which can interfere with conventional redox-based cell viability assays such as (3-(4,5-dimethyl-2-thiazolyl)-2,5-diphenyl-2H-tetrazolium bromide (MTT), leading to inaccurate or false-positive results. To avoid this, cytotoxicity was evaluated using the trypan blue exclusion assay, which directly counts viable cells without relying on metabolic activity. HUVECs and HDFa were seeded in 96-well plates at a density of 5 × 10^4^ cells per well and cultured for 48 h under standard conditions. Following this, cells were treated with varying concentrations of AP39 collected from the in vitro release study and incubated for 24 h at 37 °C with 5% CO_2_. After incubation, cells were gently harvested using trypsinisation, then mixed in a 1:1 ratio with 0.4% trypan blue dye solution. The mixture was allowed to incubate at room temperature for 3–5 min to enable dye uptake by non-viable cells with compromised membranes. Cell suspensions were then loaded onto a haemocytometer and examined under a light microscope. Viable cells remain unstained, whereas dead or damaged cells take up the dye and appear blue. Cell viability was calculated as the percentage of viable cells in relation to total cell count.

#### 2.4.2. Quantification of H_2_S Release in HUVEC and HDFa Cells

Free H_2_S reduces the tetrazolium dye MTT (Sigma, St. Louis, MO, USA) to form a purple formazan product. This reaction was utilised to quantify H_2_S release from AP39 formulations, adapting a previously described method [31]. A section of the hydrogel was carefully cut to contain an amount of AP39 that would not exceed a concentration of 300 nM in the release medium, assuming complete release over 24 h. The hydrogel samples were placed directly into wells of a 12-well plate containing pre-seeded cells and cell culture medium. At specified time points over 24 h, 100 µL of culture medium was removed and combined with an equal volume of MTT solution (5 mg/mL). Subsequently, the mixture was incubated for 3 h in a humidified environment at 37 °C, with 5% CO_2_ thus maintaining cell culture conditions and limiting evaporation. Finally, absorbance was measured at 570 nm in a microplate reader. Serial dilutions of freshly prepared sodium sulphide were used to prepare a calibration curve enabling conversion of absorbance changes into H_2_S concentrations.

#### 2.4.3. Measurement of Intracellular ROS in Cell Cultures

The intracellular ROS generated were assessed using the Cellular ROS Assay Kit (DCFDA/H2DCFDA, Abcam, Cambridge, UK, Cat. #ab113851) following the instructions, with slight modifications, as previously described [29]. HUVECs and HDFa were seeded at 1 × 10^4^ cells/well in clear-bottom, black-walled 96-well plates. Following cell stimulation with TNF-α (2.5 ng/mL) for 3 h, the media were replaced with standard media, and cells were incubated for 24 h to establish an inflammatory baseline as described previously. They were then treated with or without AP39 gel formulation permeate diluted to 0.3 µM for 24 h [32]. Cells were washed with PBS and incubated with 25 µM DCFDA in serum-free medium for 50 min at 37 °C, protected from light. Fluorescence was evaluated at wavelengths of 485/535 nm with a Spark^®^ microplate reader (TECAN, Männedorf, Switzerland).

#### 2.4.4. Assessment of Mitochondrial Membrane Potential (ΔΨm) Using TMRM Staining in HUVEC and HDFa Cells

Mitochondrial membrane potential was assessed using the potentiometric dye tetramethylrhodamine methyl ester (TMRM; Invitrogen, Waltham, MA, USA). Following exposure to TNFα as described in Section 2.4.3 and treatment with or without AP39 hydrogel permeate (0.3 µM) for 24 h, the culture medium was removed, followed by a cell wash with PBS. Next, 50 µL of TMRM solution was added, and cells were incubated in the dark for 30 min, again at 37 °C. Then, the medium was removed, and cells were washed in HEPES buffer. Fluorescence imaging was completed with a microscope. To quantify fluorescence intensity, a Spark^®^ microplate reader at 548/573 nm (TECAN, Männedorf, Switzerland) was used.

#### 2.4.5. In Vitro Investigation of Inflammatory Modulation and Extracellular Matrix Remodelling

To investigate the anti-inflammatory activity and matrix-regulatory effects of AP39 permeate exposure, the secretion of interleukin-6 (IL-6), interleukin-8 (IL-8), matrix metalloproteinase-9 (MMP-9), and transforming growth factor-β (TGF-β) was performed following quantification in cell culture supernatants using Human DuoSet ELISA kits (R&D Systems; Cat. #DY202, #DY208, #DY911, and #DY240, respectively) as reported previously [29]. HUVECs and HDFa cells were plated in 48-well plates and allowed to adhere overnight under standard culture conditions. An inflammatory phenotype was caused by treatment with TNF-α as described in Section 2.4.3. Cells were subsequently washed with PBS and exposed to AP39 gel permeate (0.3 µM) for 24 h. Following treatment, conditioned media were removed and stored at −80 °C, awaiting analysis with ELISA according to the manufacturers’ instructions.

#### 2.4.6. Assessment of the Wound Healing Capacity of AP39 Using a Scratch Assay in HUVECs

The pro-migratory and wound healing effects of AP39 were evaluated using an in vitro scratch assay in HUVECs. HUVECs were selected to specifically evaluate endothelial cell migration, an essential process in angiogenesis during wound repair. Cells were plated into 24-well plates at 5.0 × 10^4^ cells per well and cultured until a confluent monolayer was established. With the exception of untreated control wells, cells were stimulated with TNF-α as described previously. A scratch was then introduced into the cell monolayer with a 200 μL pipette tip (defined as 0 h), and two washes with PBS were performed to remove unattached cells and cellular debris [33]. Cells were subsequently treated with AP39 permeate (0.3 µM) for 24 h. Wound closure was imaged at 24 h using a Nikon Eclipse Ti-E phase-contrast inverted microscope (Nikon Instruments Inc., Melville, NY, USA). The extent of wound closure was measured with the ImageJ Version 1.54i Wound Healing Tool plugin (Montpellier Resources Imagerie, Montpellier, France) and expressed as a percentage in relation to the primary wound area.

#### 2.4.7. Tube Formation Assay in HUVECs Treated with AP39

Endothelial angiogenic activity was assessed using an in vitro tube formation assay performed on growth factor–reduced Matrigel (Corning) in 24-well plates [34]. HUVECs were seeded onto Matrigel-coated surfaces and stained with Calcein AM (Thermo Fisher) to facilitate the visualisation of capillary-like network formation. Following incubation, tube formation was evaluated by capturing images from four randomly selected fields per well at 200× magnification using a Nikon inverted microscope. Next, the total tube length was quantified with the ImageJ Angiogenesis Analyzer plugin.

### 2.5. Statistical Methods and Data Analysis

All quantitative data are presented as mean ± standard deviation (SD). Statistical analyses were conducted using GraphPad Prism (version 9.4.1; GraphPad Software, La Jolla, CA, USA). Group comparisons were performed using either unpaired or paired Student’s *t*-tests or one- and two-way analysis of variance (ANOVA), with appropriate post hoc testing applied where required. A *p*-value below 0.05 was considered indicative of statistical significance.

## 3. Results

### 3.1. Dynamic Rheological Behaviour of the Hydrogel Formulations

Hydrogel formulations containing 0.02% (*w*/*w*) AP39 and corresponding blank controls were successfully prepared, exhibiting uniform structure and optical translucency (Figure 1a). The mechanical stability of the hydrogel network was assessed through rheological analysis, focusing on elastic (storage) modulus (G′), viscous (loss) modulus (G″), and complex viscosity (η*). G′ provides an indication of the material’s solid-like behaviour, whereas G″ reflects its liquid-like response and η* describes the general resistance of the hydrogel to deformation when subjected to oscillatory shear. The G′ of the hydrogels was evaluated at 5 Hz and 30 Hz to assess the AP39 incorporation effect on gel stiffness (Figure 1b). At 5 Hz, the blank hydrogels (0% *w*/*w* AP39) exhibited a mean G′ of 5532 ± 977 Pa, while AP39-loaded hydrogels (0.02% *w*/*w*) demonstrated a comparable value of 6070 ± 653 Pa. At 30 Hz, G′ increased as expected, with values of 18,543 ± 1301 Pa and 17,300 ± 400 Pa for unloaded and loaded gels, respectively. This increase at a higher frequency is attributed to limited molecular relaxation, which causes the gel network to resist deformation and behave more elastically. These results indicate that incorporation of AP39 at 0.02% *w*/*w* does not significantly alter the elastic properties of the hydrogel, and that both formulations maintain frequency-dependent solid-like behaviour suitable for wound dressing applications.

The viscous modulus G″ of formulations containing 0% *w*/*w* and 0.02% *w*/*w* AP39 was measured at frequencies of 5 Hz and 30 Hz (Figure 1c). At 5 Hz, the control sample (0% AP39) exhibited an average G″ of 5390 ± 343 Pa, while the 0.02% AP39 formulation showed a slightly lower value of 5340 ± 319 Pa. At 30 Hz, G″ increased for both samples, with the control formulation displaying 20,000 ± 2217 Pa and the 0.02% AP39 sample showing a lower viscous modulus of 15,000 ± 1338 Pa.

Figure 1d shows the complex viscosity (η*) of hydrogels containing 0% and 0.02% *w*/*w* AP39 across increasing frequencies, demonstrating a characteristic shear-thinning profile. With increasing frequency, η* reduced substantially for both formulations, indicating a lower viscosity under higher shear conditions. This shear-thinning behaviour is advantageous for hydrogel applications, allowing for easier application under stress while maintaining structural integrity afterward. At 0.5 Hz, the unloaded formulation (0% *w*/*w* AP39) exhibited a complex viscosity of 415 ± 100 Pa·s, which decreased to 80 ± 27 Pa·s at 30 Hz. In comparison, the 0.02% *w*/*w* AP39 formulation showed a higher initial complex viscosity of 607 ± 180 Pa·s at 0.5 Hz, reducing to 54.2 ± 11.0 Pa·s at 30 Hz. The 0.02% AP39 formulation consistently exhibited higher η* values at lower frequencies, though both formulations followed a similar decreasing trend. These results indicate that the inclusion of AP39 modestly increases complex viscosity at low frequencies without significantly altering the overall shear-thinning behaviour of the system.

### 3.2. Adhesion Properties of Formulated Hydrogels

The adhesive properties of the hydrogels were evaluated with a ball-tack test, which measures the detachment force needed to separate the gel from a rigid substrate, thereby modelling practical application and removal conditions. The test consisted of two stages: a loading phase, during which a 1-inch steel probe was lowered into contact with the hydrogel surface, and an unloading phase, where the probe was retracted until complete detachment occurred. A representative force-displacement profile is provided in Supplementary Figure S1. The peak tensile force recorded during unloading corresponds to the maximum pull-off force and is a measure of adhesive strength. For the AP39 formulations, there was no statistically significant difference in maximum pull-off force between the control (0% *w*/*w* AP39: 2.60 ± 0.14 gf) and the 0.02% *w*/*w* AP39 hydrogel (2.35 ± 0.20 gf), suggesting that incorporation of AP39 at this concentration does not adversely affect the adhesive strength of the hydrogel (Table 1).

The minimum force documented in the loading phase reflects compressive resistance encountered as the probe makes contact with and compresses the hydrogel surface. This measure indicates the extent of surface adaptation by the hydrogel. The recorded minimum forces were −3.02 ± 0.10 gf for the control gel and −2.91 ± 0.14 gf for the 0.02% *w*/*w* AP39 gel (Table 1). These results similarly show no significant difference, suggesting incorporation of AP39 does not alter the hydrogel’s surface conformity or initial contact resistance.

### 3.3. Long-Term Stability of AP39 in Hydrogel

**Figure 2:**
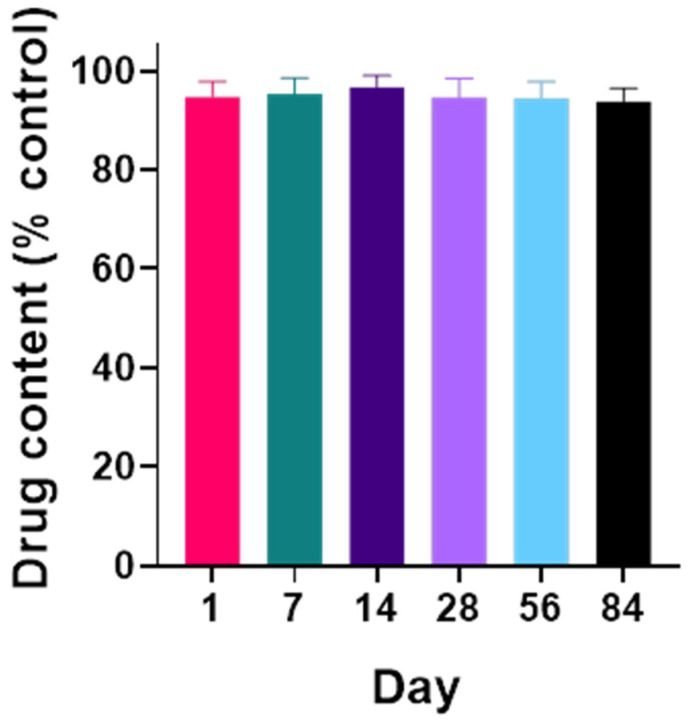
AP39 was successfully incorporated into Na-AMPS hydrogels and remained chemically stable for at least 84 days. Quantification of AP39 content over 84 days confirmed its chemical stability within the hydrogel matrix. Samples were homogenised in acetonitrile using ceramic beads and centrifuged, and the supernatant was analysed by HPLC-UV. Drug levels remained consistent with the theoretical loading throughout the study. Data represent mean ± SD, *n* = 6. Comparisons between groups were performed using one-way ANOVA.

The stability of AP39 within the hydrogel formulation was maintained over an 84-day storage period under controlled conditions (25 °C, 60 % RH). This assessment was performed to ensure the suitability of the dressing for clinical and commercial application, where prolonged shelf stability is essential, particularly for chronic wound care in diabetes. Drug content remained consistently above 93% of the initial loading, with mean values ranging from 94.7% on Day 1 to 93.8% on Day 84 (Figure 2). Variability was minimal across time points, and no statistically significant differences were observed in drug content compared to the initial measurement. These results indicate that the hydrogel matrix effectively preserved AP39 stability throughout the test period, supporting its potential for long-term storage and clinical use.

### 3.4. Hydrogel Swelling Characteristics and AP39 Release Profile

**Figure 3:**
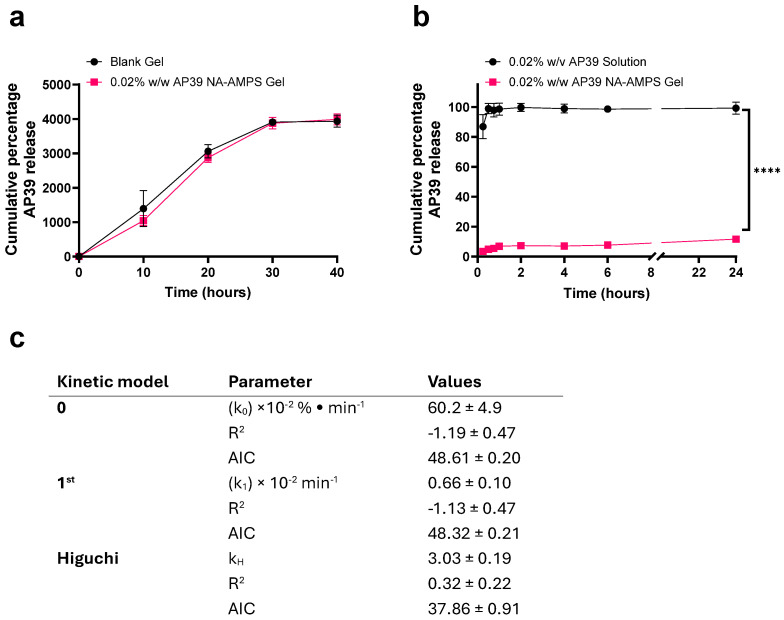
(**a**) Time-dependent water uptake of Na-AMPS hydrogels with and without 0.02% (*w*/*w*) AP39 over 40 min. Both formulations reached their swelling equilibrium by 30 min, showing comparable hydration profiles. (**b**) Cumulative in vitro release of AP39 from the hydrogel matrix compared to aqueous solution over 24 h, measured using a dialysis system. (**c**) In vitro release kinetics models: R^2^, coefficient of determination; AIC, Akaike Information Criterion; F, fraction of drug released at time t; k0, zero-order release constant; hydrogels were prepared from a Na-AMPS polymer matrix with propylene glycol and Poloxamer 184, incorporating AP39 at 0.02% (*w*/*w*), and cross-linked via photopolymerisation. Data represent mean ± SD, *n* = 4. Comparisons were performed using paired Student’s *t*-tests. Statistical significance: **** *p* < 0.0001.

The swelling behaviour of both blank and AP39-loaded Na-AMPS hydrogels was monitored over a 40 min period in water at ambient temperature (Figure 3a). Rapid hydration was observed for both formulations during the initial 10 min, followed by a more gradual increase as the gels approached equilibrium. Peak swelling occurred at 30 min, reaching 3908.2 ± 101.4% for the blank hydrogel and 3877.1 ± 165.9% for the AP39-loaded formulation, with no statistically significant difference detected between the groups (*p* > 0.05). Beyond this time point, swelling levels stabilised, with minimal variation observed at 40 min, confirming attainment of equilibrium. Overall, these findings indicate that loading the hydrogel with 0.02% *w*/*w* AP39 did not adversely affect its water uptake properties.

The release of AP39 from the solution and hydrogel formulations into PBS/ethanol (90:10) over 24 h was quantified and expressed as a percentage of the total drug content (Figure 3b). The solution showed near-complete release, with 99.2 ± 4.0% of AP39 released after 24 h. In contrast, the hydrogel released only 11.6 ± 1.2% over the same period, demonstrating a markedly sustained release profile. Cumulative AP39 release was fitted to zero-order, first-order, and Higuchi models. Both zero- and first-order models showed poor fits (negative R^2^ and higher AIC), whereas the Higuchi model provided the best fit (R^2^ = 0.32 ± 0.22, AIC = 37.86 ± 0.91), indicating that release is predominantly diffusion-controlled through the hydrogel matrix. This aligns with the observed slow, sustained release profile.

### 3.5. Evaluation of Cell Viability Following Exposure to AP39-Containing Hydrogel Permeates

**Figure 4:**
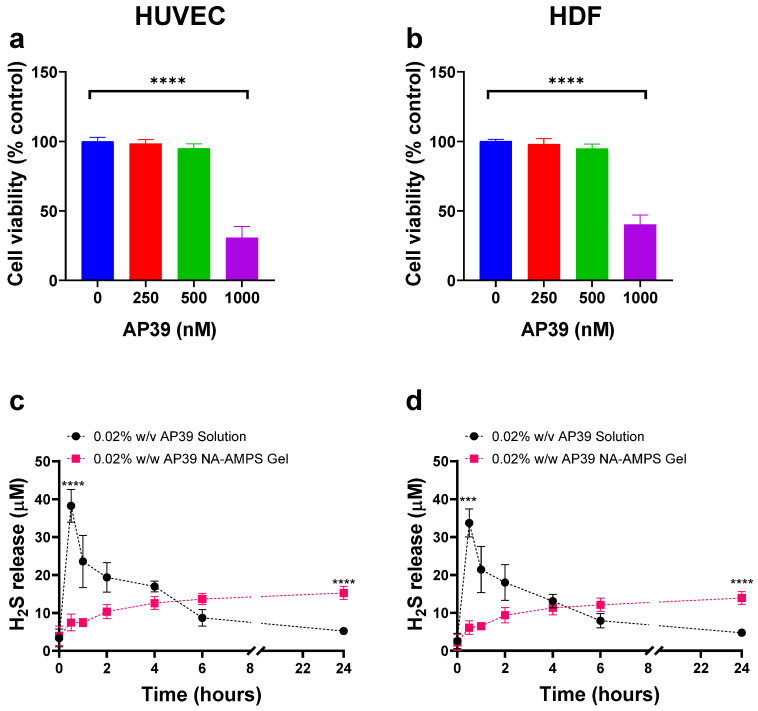
Cell viability of HUVECs (**a**) and HDFa (**b**) following 24 h exposure to hydrogel permeate containing varying concentrations of AP39. Cell viability was assessed following trypan blue staining and cell counting. H_2_S release into the culture medium was assessed in HUVECs (**c**) and HDFa (**d**) over 24 h of AP39 either in solution or gel format (500 nM maximum theoretical dose). Data represent mean ± SD, *n* = 6. Comparisons were performed using unpaired Student’s *t*-tests or one-way ANOVA with Tukey’s post hoc test. Statistical significance: *** *p* < 0.001, **** *p* < 0.0001.

Cell viability of HUVECs following 24 h exposure to increasing concentrations of AP39 collected from the in vitro release study (0–1000 nM) was assessed. As shown in Figure 4a, AP39 was well tolerated at concentrations up to 500 nM, with viability remaining above 95%. Specifically, cell viability was 100.1 ± 2.9% at 0 nM, 98.5 ± 2.9% at 250 nM, and 95.1 ± 3.1% at 500 nM. However, a marked reduction in viability was observed at 1000 nM, where viability dropped to 30.7 ± 8.1%, indicating concentration-dependent cytotoxicity at higher doses. These findings suggest that AP39 is non-cytotoxic to HUVECs at concentrations less than 500 nM, supporting its use at this concentration or lower in subsequent studies.

Cell viability of HDFa cells following 24 h exposure to AP39 (0–1000 nM) is presented in Figure 4b. Similarly to HUVECs, AP39 showed no significant cytotoxicity at concentrations up to 500 nM. Viability was 100.3 ± 1.3% at 0 nM, 98.2 ± 3.9% at 250 nM, and 94.9 ± 3.2% at 500 nM. However, at 1000 nM, viability was reduced to 40.2 ± 6.8%, indicating a decrease in cell viability at higher doses. Similarly to results seen with HUVECs, data suggest AP39 is well tolerated by HDFa cells at concentrations less than 500 nM.

Furthermore, cell viability of HaCaT following 24 h exposure to AP39 (0–1000 nM) was investigated (Supplementary Figure S2). HaCaT were included in the viability assessment to evaluate cytocompatibility at the wound periphery, as the adhesive hydrogel is intended to contact and adhere to healthy epidermal tissue surrounding the wound site. HaCaT viability remained comparable to untreated controls at lower concentrations of AP39, with values of 100.7 ± 2.4% at 0 nM and 99.4 ± 2.8% at 250 nM. A non-significant reduction in viability was observed at 500 nM (94.7 ± 4.5%). In contrast, exposure to 1000 nM AP39 resulted in a pronounced reduction in cell viability to 31.2 ± 4.4%, demonstrating a clear cytotoxic effect at higher doses. Consistent with observations in HUVECs and HDFa cells, these findings indicate AP39 is well tolerated by HaCaT at concentrations up to 500 nM, with significant cytotoxicity evident at 1000 nM.

### 3.6. Quantification of H_2_S Release from AP39 Hydrogels in HUVEC and HDFa Cells

The release of H_2_S from both AP39 solution and AP39-loaded hydrogel formulations (0.3 µM) was measured at over 24 h in both HUVEC and HDFa cells (Figure 4c and Figure 4d, respectively). In HUVECs, at 0.5 h, the AP39 solution exhibited a significantly higher H_2_S release, with a mean concentration of 38.2 ± 4.4 µM, compared to 7.45 ± 2.21 µM from the hydrogel formulation (*p* < 0.0001). However, over 24 h the hydrogel showed a sustained release of H_2_S with 15.3 ± 1.7 µM observed at 24 h, which was notably greater than the 5.2 µM ±0.6 detected from the solution (*p* < 0.0001). These results indicate that the hydrogel formulation provides a controlled, sustained release of H_2_S over time, in contrast to the rapid release observed with the solution. In HDFa cells, H_2_S release from the solution and hydrogel formulations showed a similar trend to that observed in HUVECs, although overall concentrations detected were slightly lower. At 1 h, the solution released 33.7 ± 3.7 µM of H2S compared to 6.1 ± 1.8 µM from the hydrogel. After 24 h, the solution concentration decreased to 4.7 ± 0.5 µM, while the hydrogel released 13.9 ± 1.7 µM. Notably, in both cell types, residual drug was still detected in the hydrogel at 24 h, indicating that complete release of AP39 was not achieved within this timeframe.

### 3.7. Measurement of Intracellular ROS in HUVEC and HDFa Cells

**Figure 5:**
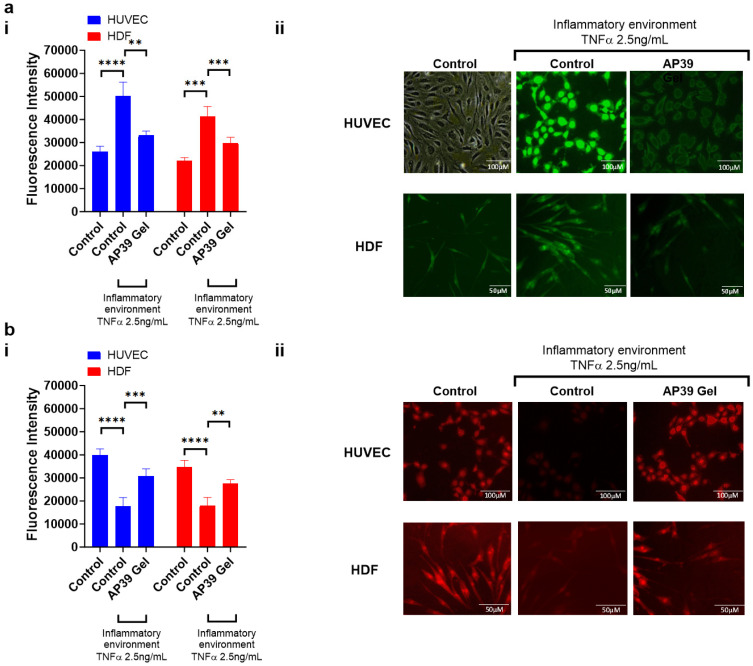
AP39-loaded hydrogels attenuate oxidative stress triggered by TNF-α and mitochondrial dysfunction in HUVEC and HDFa cells. (**a**) (i) Quantification of intracellular ROS fluorescence intensity and (**a**) (ii) representative fluorescence images. (**b**) (i) Mitochondrial membrane potential, assessed via TMRM fluorescence, and (**b**) (ii) representative images. Data represent mean ± SD *n* = 6. Comparisons were performed using one-way ANOVA with Tukey’s post hoc test. Statistical significance: ** *p* < 0.01, *** *p* < 0.001, **** *p* < 0.0001.

To determine whether AP39 (0.3 µM) could reduce oxidative stress in endothelial cells, intracellular ROS levels were measured following TNF-α stimulation (Figure 5a). ROS levels increased significantly from 26,162 ± 2203 AU in the untreated control to 50,122 ± 5999 AU after TNF-α treatment (*p* < 0.0001). Treatment with AP39 reduced ROS levels to 33,087 ± 1865 AU, which was significantly lower than the TNF-α-only group (*p* < 0.0001), indicating that AP39 effectively mitigates TNF-α-induced oxidative stress in HUVECs. ROS levels were also assessed in HDFa cells under TNF-α stimulation. ROS levels increased from 22,183 ± 1294 AU in control cells to 41,367 ± 4225 AU following TNF-α exposure (*p* < 0.0001). AP39 treatment reduced ROS levels to 29,813 ± 2406 AU, which was significantly lower than the TNF-α-only group (*p* < 0.0001), demonstrating that AP39 attenuates oxidative stress in inflamed fibroblasts.

### 3.8. Mitochondrial Membrane Potential (ΔΨm) Assessed by TMRM Fluorescence

To evaluate the effect of AP39 (0.3 µM) on mitochondrial health under inflammatory stress, TMRM fluorescence intensity was measured in HUVEC and HDFa cells treated with TNF-α, with or without AP39 (Figure 5b). In HUVECs, exposure to TNF-α led to a significant reduction in mitochondrial membrane potential compared to untreated controls (17,706 ± 3773 vs. 39,993 ± 2625 AU; *p* < 0.0001). Subsequently, treatment with 0.3 µM AP39 significantly restored TMRM fluorescence (30,833 ± 3056 AU; *p* < 0.001 vs. TNF-α alone), indicating a protective effect on mitochondrial function. A similar pattern was observed in HDFa cells, where TNF-α treatment caused a significant drop in TMRM signal relative to controls (17,994 ± 3535 vs. 34,775 ± 2835 AU; *p* < 0.0001). AP39 treatment partially rescued the membrane potential, with fluorescence intensity increasing to 27,661 ± 1613 AU (*p* < 0.01 vs. TNF-α). These findings suggest that AP39 preserves mitochondrial membrane potential in both endothelial and dermal fibroblast cells under pro-inflammatory conditions.

### 3.9. Assessment of Anti-Inflammatory and Matrix-Modulating Effects of AP39-Loaded Hydrogel in HUVEC and HDFa Cells

**Figure 6:**
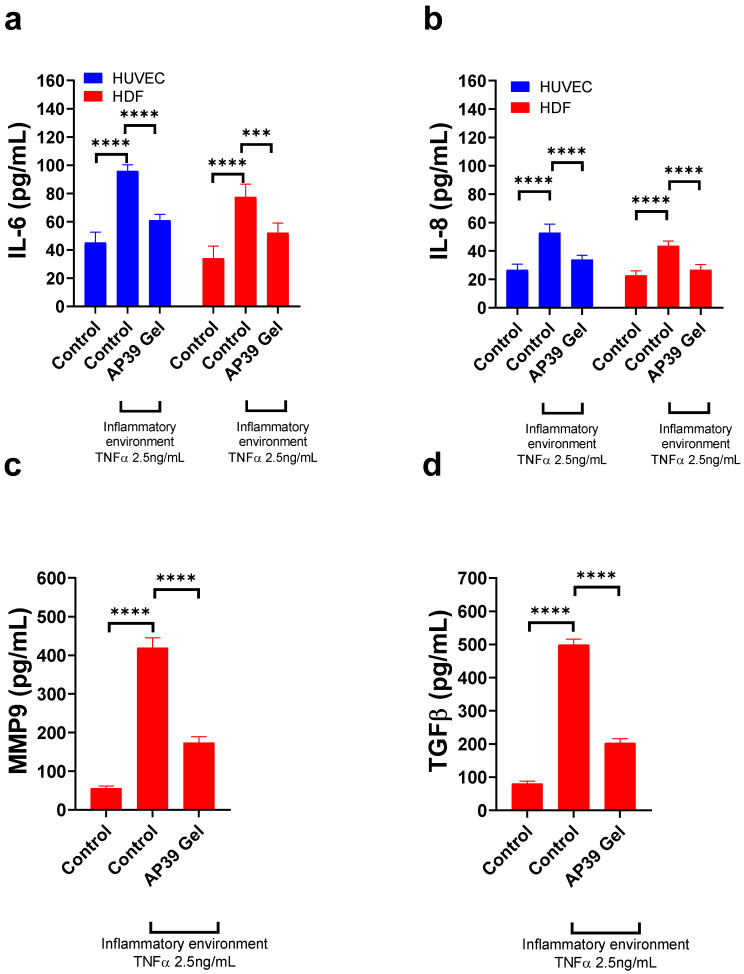
Effects of AP39 hydrogel permeate on inflammatory and matrix-related responses in HUVEC and HDFa cells. (**a**) IL-6 and (**b**) IL-8 secretion were markedly elevated in both cell types following TNF-α exposure (2.5 ng/mL), and treatment with AP39 hydrogel significantly attenuated these cytokine levels. (**c**) In HDFa cells, TNF-α-induced MMP-9 secretion was significantly reduced upon AP39 hydrogel treatment. (**d**) TGF-β secretion increased under inflammatory conditions and was moderately suppressed by AP39 hydrogel. Data are presented as mean ± SD, *n* = 4. Comparisons were performed using one-way ANOVA with Tukey’s post hoc test. Statistical significance: *** *p* < 0.001, **** *p* < 0.0001.

To evaluate the potential anti-inflammatory effects of AP39 (0.3 µM), IL-6 secretion was measured in HUVECs stimulated with TNF-α, a key pro-inflammatory cytokine. As shown in Figure 6a, IL-6 levels were significantly elevated in the TNF-α-treated group (96.1 ± 4.2 pg/mL) compared to the untreated control (45.3 ± 7.3 pg/mL), confirming the successful induction of inflammation (*p* < 0.0001). Next, treatment with 0.3 µM AP39 significantly limited IL-6 secretion to 60.9 ± 4.21 pg/mL (*p* < 0.0001), suggesting that AP39 exerts anti-inflammatory effects by attenuating TNF-α-induced IL-6 production. These findings support the potential of AP39 in modulating inflammatory responses. To assess the anti-inflammatory effects of AP39 HDFa, IL-6 secretion was measured following TNF-α stimulation. IL-6 levels increased significantly from 34.1 ± 8.5 pg/mL in the untreated control to 77.5 ± 8.9 pg/mL with TNF-α treatment, confirming successful induction of inflammation (*p* < 0.0001). Treatment with 0.3 µM AP39 reduced IL-6 levels to 52.3 ± 6.8 pg/mL, which was significantly lower than the TNF-α-only group (*p* < 0.001), indicating that AP39 attenuates TNF-α-induced IL-6 production and may exert anti-inflammatory effects in HDFa cells.

To supplement IL-6 measurements and further investigate the anti-inflammatory effects of AP39 in HUVECs, IL-8 secretion was measured following TNF-α stimulation (Figure 6b). IL-8 levels increased significantly from 26.7 ± 3.9 pg/mL in untreated control cells to 52.9 ± 5.9 pg/mL after TNF-α treatment, confirming successful induction of inflammation (*p* < 0.0001 vs. control). Treatment with AP39 reduced IL-8 levels to 33.9 ± 3.0 pg/mL, which remained significantly lower than those in the TNF-α-only group (*p* < 0.0001), suggesting that AP39 effectively attenuates TNF-α-induced IL-8 production in endothelial cells. Anti-inflammatory effects of AP39 in human dermal fibroblasts were also investigated. IL-8 secretion was measured following TNF-α stimulation. IL-8 levels increased significantly from 22.8 ± 3.2 pg/mL in the untreated control to 43.7 ± 3.4 pg/mL after TNF-α treatment, confirming successful induction of inflammation (*p* < 0.0001 vs. control). Treatment with AP39 reduced IL-8 levels to 26.7 ± 3.7 pg/mL, which was significantly lower than the TNF-α-only group (*p* < 0.0001), suggesting that AP39 can attenuate TNF-α-induced IL-8 production in dermal fibroblasts.

To further evaluate the anti-inflammatory and matrix-modulating effects of AP39 in human dermal fibroblasts, MMP-9 secretion was measured following TNF-α stimulation (Figure 6c). TNF-α treatment markedly increased MMP-9 levels from 56.3 ± 5.4 pg/mL in untreated control cells to 419.4 ± 25.5 pg/mL, indicating robust inflammatory activation (**** *p* < 0.0001). Next, treatment with AP39 reduced MMP-9 secretion to 174.0 ± 15.1 pg/mL, which remained significantly lower than in the TNF-α-only group (**** *p* < 0.0001). These data suggest AP39 effectively suppresses TNF-α-induced MMP-9 production, supporting its potential role in modulating extracellular matrix remodelling during inflammation.

To assess the impact of AP39 on profibrotic signalling in human dermal fibroblasts, TGF-β secretion was measured following TNF-α stimulation (Figure 6d). TNF-α markedly increased TGF-β levels from 80.3 ± 7.7 pg/mL in the untreated control to 499.3 ± 16.8 pg/mL, confirming significant upregulation of fibrotic signalling pathways (**** *p* < 0.0001). Treatment with AP39 reduced TGF-β levels to 203.3 ± 12.6 pg/mL, significantly lower than the TNF-α-only group (**** *p* < 0.0001), suggesting AP39 may suppress inflammation-associated profibrotic responses in dermal fibroblasts.

### 3.10. Effects of AP39 on Endothelial Angiogenesis and Wound-Related Migration

**Figure 7:**
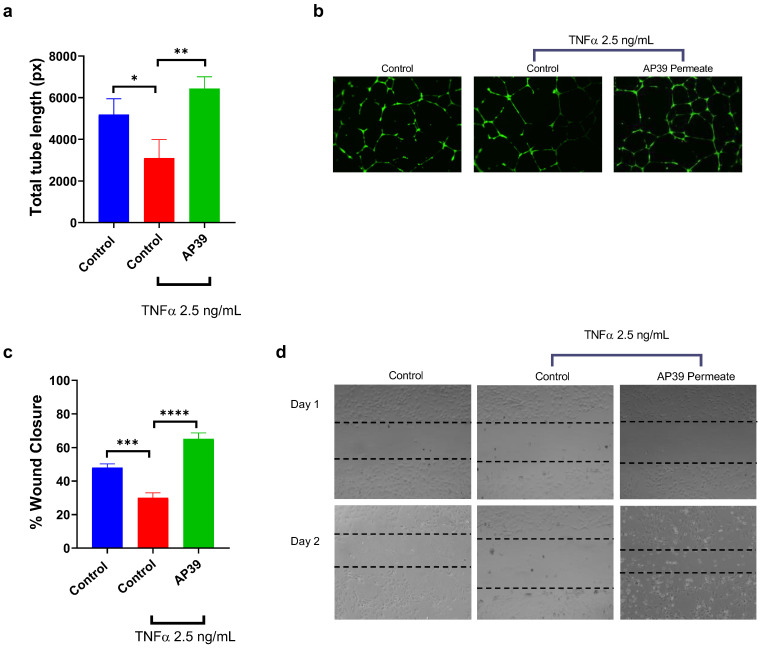
AP39 permeate restores TNF-α–impaired cell migration in the scratch wound healing assay as well as angiogenesis. (**a**) Quantification of total tube length (px) in HUVECs under control conditions, following TNF-α stimulation (2.5 ng/mL), and after treatment with AP39. (**b**) Representative images of endothelial tube formation for each experimental condition, illustrating network complexity and vessel-like structure organisation. (**c**) Quantitative analysis of percentage wound closure. (**d**) Representative images of scratch wounds at 0 h and 24 h for control, TNF-α, and ADT-OH + TNF-α groups. Data represent mean ± SD, *n* = 3. Comparisons were performed using one-way ANOVA with Tukey’s post hoc test. Statistical significance is indicated as follows: * *p* < 0.05, ** *p* < 0.01, *** *p* < 0.001; **** *p* < 0.0001.

HUVECs were utilised to investigate endothelial cell behaviour relevant to wound healing, with angiogenic capacity first assessed using a tube formation assay, followed by evaluation of endothelial migration using a scratch assay. Total tube length analysis of HUVEC cell cultures from the angiogenesis assay demonstrated a marked reduction in network formation following inflammatory stimulation with TNF-α (2.5 ng/mL) compared with the untreated control (Figure 7a,b). The control group exhibited a mean total tube length of 5190 ± 758 px, which was significantly decreased to 3095 ± 894 px in TNF-α–treated cultures (*p* < 0.05), indicating impaired angiogenic capacity under inflammatory conditions. Treatment with 0.3 µM AP39 markedly enhanced tube formation, yielding a mean total tube length of 6432 ± 571 px (*p* < 0.01). This represented a substantial increase relative to the TNF-α–stimulated group and exceeded levels observed in the untreated control, suggesting that AP39 not only mitigated inflammation-induced angiogenic impairment but also promoted endothelial network formation.

The scratch wound healing assay demonstrated that exposure to TNF-α significantly reduced cell migration, with the percentage of wound closure decreasing from 48.0 ± 2.2% in the untreated control to 30.1 ± 2.9% (*p* < 0.001 vs. control) (Figure 7c,d). Treatment with AP39 following TNF-α exposure resulted in a marked increase in wound closure to 65.1 ± 3.6%, which was significantly greater than both the TNF-α group (**** *p* < 0.0001) and the untreated control (*p* < 0.001). These results indicate that AP39 effectively mitigates TNF-α-induced impairment of cell migration, promoting enhanced wound repair.

## 4. Discussion

The design of advanced hydrogel wound dressings demands integration of structural robustness, effective adhesion to tissue, controlled release of therapeutics, and biological compatibility to actively support tissue regeneration. In recent years, H_2_S has emerged as a promising therapeutic mediator in wound healing due to its roles in redox regulation, inflammation modulation, and angiogenesis [35]; however, its clinical translation is limited by its volatility and short biological half-life [36]. Controlled delivery systems are therefore required to harness its therapeutic potential. In this study, a Na-AMPS-based adhesive hydrogel incorporating AP39, a mitochondria-targeted H_2_S donor with antioxidant activity, was developed and systematically evaluated as a bioactive wound dressing platform. Physicochemical and functional characterisation encompassed rheological behaviour, adhesive performance, swelling and release characteristics, as well as in vitro cytocompatibility. Biological efficacy was further assessed through analysis of inflammatory mediators and functional wound healing–relevant assays. Collectively, findings demonstrate this hydrogel system enables sustained, localised H_2_S delivery while maintaining favourable mechanical integrity and biological performance, supporting its potential application as a therapeutic wound dressing.

The hydrogels appeared visually homogeneous, and AP39-loaded formulations were uniformly translucent, indicating consistent drug incorporation and a stable polymer network. These observations suggest that the hydrogel matrix allows uniform distribution of the active compound without visible phase separation or aggregation. The translucency and smooth appearance further imply a well-formed network that could support controlled drug release and maintain mechanical integrity during application. Although these observations indicate a uniform network, further physicochemical characterisation, such as FT-IR to confirm hydrogel–drug interactions and SEM to evaluate morphology, could provide additional mechanistic insight and will be considered in future studies.

Analysis of the storage (G′) and loss (G″) moduli provides insight into the viscoelastic behaviour of hydrogel systems, where G′ reflects elastic, solid-like mechanical stability and G″ represents viscous, liquid-like flexibility, both of which are essential for effective adhesive wound dressing performance. For such applications, a predominantly elastic response is required to preserve structural integrity and adhesion when subjected to external forces, while a viscous component contributes to material conformability and user comfort [37,38]. In the present study, both blank and AP39-loaded hydrogels exhibited storage moduli that consistently exceeded loss moduli across the tested frequency range, indicating a dominant elastic response and a solid-like network structure. This mechanical profile supports the suitability of the formulation for adhesive applications where shape retention and durability are required. Comparable rheological profiles have been reported for other polysaccharide-based and composite hydrogels developed for wound healing applications, where frequency-independent dominance of G′ has been associated with network robustness and resistance to deformation during handling and wear [39]. Similarly, hyaluronic acid–based hydrogels functionalised with dopamine or imidazole have demonstrated storage moduli exceeding loss moduli across relevant frequency ranges, supporting their mechanical stability for wound dressing applications [40]. Importantly, incorporation of AP39 did not adversely affect the elastic dominance of the hydrogel. The relatively narrow separation between G′ and G″ observed suggests a balanced viscoelastic response, which may enhance spreadability and comfort upon application. However, this balance may also limit resistance to prolonged or dynamic mechanical loading. As such, future optimisation could focus on increasing elastic dominance through adjustments to crosslinking density, polymer composition, or incorporation of reinforcing components, while maintaining biocompatibility and controlled drug release performance.

Across the frequency range examined, both the G′ and loss G″ moduli increased with rising oscillatory frequency, indicating enhanced resistance of the hydrogel network to deformation under dynamic loading. Introduction of AP39 at 0.02% *w*/*w* led to a modest decrease in both moduli, consistent with a slight softening of the polymer network; however, the overall viscoelastic behaviour remained unchanged, with elastic responses continuing to lead. This suggests that incorporation of the active compound did not compromise the mechanical integrity or adhesive functionality of the hydrogel. In addition, both blank and AP39-loaded formulations displayed clear shear-thinning behaviour, demonstrated by a marked reduction in complex viscosity as frequency increased. This characteristic is advantageous for topical application, allowing the hydrogel to temporarily flow under applied stress during spreading or repositioning, while rapidly recovering viscosity once the stress is removed to maintain adhesion at the wound site. Comparable frequency-dependent viscosity decreases have been reported in other biomedical hydrogel systems, where it is essential for processability while maintaining structural stability under physiological conditions [41]. Such viscoelastic profiles reflect a critical balance between mechanical integrity and functional performance. Notably, the slightly higher viscosity observed at low frequencies for the AP39-loaded hydrogel may further enhance handling properties by providing a firmer, yet still compliant, material.

Adhesion is a key functional requirement for wound dressings, as the material must remain securely positioned at the wound site while allowing atraumatic removal to minimise patient discomfort and tissue damage. In this study, adhesive behaviour was evaluated using a ball-tack assay, which enables quantitative assessment of both the force required for detachment and the initial contact force during application. The results showed that incorporation of AP39 at 0.02% *w*/*w* did not result in a significant change in maximum pull-off force when compared with the unloaded hydrogel, indicating that therapeutic loading at this level does not adversely affect the adhesive strength. In addition, the force measured during the loading phase, which reflects the ability of the hydrogel to establish conformal contact with the substrate, was comparable between formulations. This suggests inclusion of AP39 does not impair surface wetting or initial adhesion, both of which are essential for effective dressing placement and sustained drug delivery [42].

The Na-AMPS–based hydrogels exhibited rapid hydration, reaching equilibrium swelling within 30 min, resulting in a highly hydrated polymer network capable of effective water uptake and retention—an essential characteristic for wound dressing applications. Comparable swelling kinetics, with full hydration occurring within 30 min, have previously been reported for Na-AMPS hydrogel systems, supporting the reproducibility of this behaviour [28,43]. Formation of a stable, hydrated matrix is likely to underpin the controlled release performance observed in the present study.

The release profile obtained demonstrates that the Na-AMPS hydrogel enables sustained and regulated delivery of AP39, in contrast to the rapid and complete release observed when AP39 is administered in solution. Incorporation within the hydrogel matrix markedly reduced the release rate over a 24 h period, with no evidence of an initial burst effect. Kinetic modelling further indicated that AP39 release from the hydrogel is best described by the Higuchi model, suggesting that diffusion through the hydrated polymer network is the primary mechanism governing drug release. Therefore, drug release was proportional to the square root of time, with the release rate dependent on the square root of drug solubility, the exposed surface area, the diffusion coefficient, and inversely related to time [44]. While similar release modulation has been achieved in more complex delivery platforms—such as liposomal or multi-component gel systems—this formulation attains controlled release using a single-polymer network, highlighting the versatility and simplicity of Na-AMPS hydrogels as a delivery platform [45,46]. Sustained local delivery is particularly advantageous for H_2_S-based therapeutics, given the transient nature and short biological half-life of free H_2_S [36]. Furthermore, the relatively slow-release profile may be advantageous for wound applications, where prolonged local delivery could reduce both dosing frequency and the need for frequent dressing changes, thereby minimising trauma to the wound site. This behaviour may also help sustain submicromolar drug concentrations, which are desirable for mitochondrial-targeted H_2_S donors to minimise potential cytotoxicity while maintaining therapeutic activity. Na-AMPS hydrogels have been reported to sustain the release of antibacterial agents such as ciprofloxacin and silver sulfadiazine over 24 h, with only ~40–55% cumulative drug release depending on molecular structure [27]. This partial release profile reflects diffusion-controlled transport modulated by polymer–drug interactions, including hydrogen bonding and electrostatic binding within the sulfonated hydrogel network. Such behaviour underscores the ability of Na-AMPS matrices to retain loaded therapeutics and provide prolonged local delivery rather than rapid burst release.

Previous carriers for AP39 delivery have included liposomal systems, polymeric nanoparticles, cellulose-based hydrogels or microneedle patches, which can provide controlled release but often require complex formulations, additional stabilisers, or lack adhesive properties suitable for dermal dressings [11,18,30,47,48]. Other H_2_S-releasing dressings, such as alginate sponges incorporating the pH-responsive donor JK-1, have shown promise by absorbing wound exudate and releasing H_2_S under acidic conditions to enhance angiogenesis and tissue regeneration [35]. However, these systems typically rely on multi-component donor–carrier designs and lack the integrated adhesion and mechanical stability required for a wound dressing. This highlights a gap in current H_2_S topical therapeutics and underscores the novelty of the present approach. In contrast, the Na-AMPS hydrogel used here offers a simple, single-polymer network that combines adhesive, hydrated, and mechanically robust properties while enabling sustained, localised AP39 release. In the present study, release behaviour was evaluated using a single AP39 loading selected to ensure that permeate concentrations remained within a safe range for subsequent cell-based assays. However, given the relatively slow release observed, higher initial loadings could potentially be used to increase the drug reservoir within the hydrogel. Future studies will therefore explore additional AP39 concentrations to further characterise release kinetics and diffusion behaviour. Finally, permeation through skin or wound tissue was not evaluated. Given the complex structure of wounds, meaningful permeation assessment would require in vivo models. Future work should include appropriate in vivo studies to confirm local bioavailability and tissue penetration, providing further insight into the therapeutic potential of the hydrogel system.

Evaluation of the cytotoxic potential of permeates collected during in vitro AP39 release was conducted using HUVEC, HDFa, and HaCaT to provide a comprehensive assessment of biocompatibility across key skin and vascular cell types. Across all three cell lines, high cell viability (>90%) was maintained at submicromolar AP39 concentrations (0.1–0.5 μM), indicating good tolerability within this therapeutically relevant range. In contrast, exposure to higher concentrations (≥1 μM) resulted in a clear, concentration-dependent decline in cell viability, demonstrating increased sensitivity at elevated doses. This trend is consistent with previous reports showing decreased viability at higher AP39 concentrations in SH-SY5Y and differentiated SH-SY5Y cells; however, these neuronal cells tolerated higher doses than the HUVECs and HDFa used in the present study, suggesting the endothelial and fibroblast cells may be more sensitive to AP39 exposure [49]. Findings emphasise the necessity for precise dose regulation in hydrogel-based delivery systems, particularly for topical or local applications, where sustained release at submicromolar levels is critical to minimise cytotoxic effects while preserving therapeutic efficacy.

Exposure of HUVECs and HDFa cells to hyperglycaemic and pro-inflammatory conditions led to a marked increase in intracellular ROS, reflecting the oxidative stress characteristic of diabetic pathology. Treatment with AP39 significantly attenuated ROS accumulation in both cell types, although levels did not fully revert to baseline, suggesting a partial yet physiologically meaningful restoration of redox balance. This finding aligns with prior reports demonstrating AP39’s ability to modulate cellular oxidative stress and protect against diabetes-associated cellular dysfunction [18,50]. At the mitochondrial level, TMRM staining revealed that AP39 partially restored mitochondrial membrane potential, which is typically depolarised under hyperglycaemic and inflammatory stress [51]. The observed improvement indicates that AP39 stabilises mitochondrial bioenergetics, likely by maintaining electron flow through the respiratory chain and preventing excessive electron leakage that drives ROS production [12,51,52]. Mechanistically, this effect may involve the H_2_S-mediated donation of electrons to the electron transport chain via sulphide quinone oxidoreductase, enhancing oxidative phosphorylation and ATP synthesis while limiting superoxide formation [53]. Additionally, H_2_S has been shown to upregulate endogenous antioxidant systems, including glutathione and superoxide dismutase, further mitigating oxidative damage [54]. By preserving mitochondrial integrity, AP39 may also modulate downstream redox-sensitive signalling pathways, such as NF-κB and NLRP3 inflammasome activation, thereby reducing pro-inflammatory gene expression and apoptosis [55].

AP39 effectively reduced IL-6 secretion in both HUVECs and HDFa cells under inflammatory conditions, demonstrating a pronounced anti-inflammatory effect. The suppression of IL-6 is particularly relevant, as persistent elevation of pro-inflammatory cytokines is a central driver of impaired wound healing in diabetic environments [56]. Comparable anti-inflammatory effects of AP39 have been observed in other experimental models. For example, in mouse models of lipopolysaccharide-induced airway inflammation, AP39 reduced neutrophil infiltration and suppressed pro-inflammatory cytokines including IL-6 and TNF-α [57]. Similarly, in rodent models of cerebral ischaemia, AP39 attenuated neuroinflammation by decreasing microglial activation and lowering IL-6 and TNF-α levels in affected brain regions, while also reducing neuronal injury and promoting pro-survival signalling [58]. The anti-inflammatory and antioxidant actions of AP39 were accompanied by enhanced functional outcomes in vitro. In tube formation assays, AP39-treated endothelial cells developed more extensive and interconnected capillary-like networks compared with stressed controls, reflecting improved angiogenic capacity. Similarly, scratch wound assays revealed accelerated endothelial migration under TNF-α-induced stress, further supporting a role for AP39 in promoting wound closure. These functional improvements likely arise from mitochondria-targeted modulation of redox balance, which in turn attenuates activation of redox-sensitive inflammatory pathways, including NF-κB and NLRP3 inflammasome signalling, thereby limiting cytokine overproduction and apoptotic responses [15,16,17].

In HDFa cells, AP39 also modulated matrix remodelling by significantly reducing MMP-9 levels, which were elevated under diabetic-like stress conditions. Excessive MMP-9 activity is known to degrade the extracellular matrix and impair granulation tissue formation, contributing to delayed wound repair [59]. Its attenuation by AP39 suggests a restoration of matrix balance and a dampening of pro-inflammatory signalling, consistent with prior studies linking MMP-9 suppression to anti-inflammatory and pro-healing effects [60,61]. Furthermore, while TGF-β secretion remained above baseline levels observed in unstressed controls, AP39 partially tempered this response, indicating an ability to limit excessive fibrotic signalling that can contribute to pathological scarring in chronic diabetic wounds. Collectively, these findings indicate that AP39 exerts multi-faceted protective effects, integrating mitochondrial bioenergetic support, reduction of oxidative stress, suppression of inflammatory pathways, and normalisation of matrix remodelling. This comprehensive profile highlights its potential utility in hydrogel-based or other localised delivery strategies aimed at enhancing cellular resilience and promoting effective tissue repair in chronic diabetic wounds.

These findings provide preliminary evidence that an AP39-loaded Na-AMPS hydrogel may be a promising approach for managing diabetic wounds. However, results are limited to in vitro models, and further *in vivo* investigations will be essential to evaluate therapeutic effectiveness, release kinetics, and biocompatibility within the more complex wound microenvironment. Additional optimisation of hydrogel mechanical properties may enhance clinical applicability. Given the high incidence of bacterial infection in chronic diabetic wounds, subsequent studies should also assess the formulation’s antimicrobial activity against relevant pathogens. Evaluating the hydrogel’s capacity to simultaneously support tissue repair and resist microbial colonisation will be critical for translating this system into a clinically effective wound dressing.

## 5. Conclusions

Chronic diabetic wounds present a major clinical challenge due to sustained inflammation, oxidative stress, impaired angiogenesis, and dysregulated matrix remodelling, all of which contribute to delayed healing and increased risk of infection. In this study, we developed an AP39-loaded Na-AMPS hydrogel that combines mechanical stability, strong adhesion, rapid hydration, and sustained, localised H_2_S delivery to address these complex pathophysiological features. AP39 was well tolerated by endothelial cells, fibroblasts, and keratinocytes at submicromolar concentrations, while higher doses highlighted the importance of controlled dosing. Functionally, AP39 reduced intracellular ROS and IL-6, partially restored mitochondrial membrane potential, modulated matrix remodelling via suppression of MMP-9 and tempering of TGF-β, and enhanced endothelial angiogenesis and migration. Together, these effects demonstrate that AP39 can simultaneously mitigate oxidative stress, inflammation, and aberrant matrix activity, translating into improved cellular processes critical for wound repair. These findings establish the AP39-loaded hydrogel as a promising multifunctional platform for diabetic wound healing.

## Figures and Tables

**Table 1:** Ball-tack adhesion measurements of Na-AMPS hydrogels using a 1-inch stainless steel ball. Maximum and minimum pull-off forces were obtained during unloading and loading, respectively.

Formulation (% *w*/*w* AP39 in Hydrogel)	Max Force (gf)	Min Force (gf)
0% *w*/*w* AP39	2.60 ± 0.14	−3.02 ± 0.10
0.02% *w*/*w* AP39	2.35 ± 0.2	−2.91 ± 0.14

## Data Availability

Data supporting the findings of this study are not currently publicly available owing to related ongoing research but can be requested from the corresponding author.

## Supplementary Materials

The following supporting information can be downloaded at: https://www.mdpi.com/article/10.3390/gels12030251/s1, Figure S1: A single representative force-displacement curve for an adhesive hydrogel loaded with AP39. Each curve illustrates the measurement from one sample and one time; Figure S2: Cell viability of HaCats following 24-h exposure to hydrogel permeate containing varying concentrations of AP39. Cell viability was assessed following trypan blue staining and cell counting. Data represent mean ± SD (*n* = 6). Statistical significance: **** *p* < 0.0001.

## References

[1] Holzer-Geissler J.C.J., Schwingenschuh S., Zacharias M., Einsiedler J., Kainz S., Reisenegger P., Holecek C., Hofmann E., Wolff-Winiski B., Fahrngruber H. (2022). The Impact of Prolonged Inflammation on Wound Healing. Biomedicines.

[2] Xiong Y., Chu X., Yu T., Knoedler S., Schroeter A., Lu L., Zha K., Lin Z., Jiang D., Rinkevich Y. (2023). Reactive oxygen species-scavenging nanosystems in the treatment of diabetic wounds. Adv. Healthc. Mater..

[3] Moreira H.R., Marques A.P. (2022). Vascularization in skin wound healing: Where do we stand and where do we go?. Curr. Opin. Biotechnol..

[4] Zhang P., Lu J., Jing Y., Tang S., Zhu D., Bi Y. (2017). Global epidemiology of diabetic foot ulceration: A systematic review and meta-analysis†. Ann. Med..

[5] Namgoong S., Jung S., Han S.K., Jeong S.H., Dhong E.S., Kim W.K. (2015). Risk Factors for Major Amputation in Hospitalised Diabetic Foot Patients. Int. Wound J..

[6] Pany S., Sen S.K., Prasanna G., Pati S., Pal B.B. (2021). Spectrum of Bacterial Infections Associated with Diabetic Ulcer Patients. J. Pure Appl. Microbiol..

[7] Akkus G., Sert M. (2022). Diabetic foot ulcers: A devastating complication of diabetes mellitus continues non-stop in spite of new medical treatment modalities. World J. Diabetes.

[8] Ciccone V., Genah S., Morbidelli L. (2021). Endothelium as a source and target of H_2_S to improve its trophism and function. J. Antioxid..

[9] Nour S., Imani R., Chaudhry G.R., Sharifi A.M. (2021). Skin wound healing assisted by angiogenic targeted tissue engineering: A comprehensive review of bioengineered approaches. J. Biomed. Mater. Res. Part A.

[10] Jiang L., Yang X., Zhang Y., He D., Gao Y., Lu K., Hao Y., Gao Y., Lu D., Jin X. (2023). Novel ROS-scavenging hydrogel with enhanced anti-inflammation and angiogenic properties for promoting diabetic wound healing. Biomater. Adv..

[11] Marwah M.K., Sanchez-Aranguren L., Shokr H., Tahan M.A.A., Wang K., Ahmad S. (2026). Advancing therapeutics with targeted formulations of hydrogen sulphide donors. Eur. J. Pharm. Sci..

[12] Szczesny B., Módis K., Yanagi K., Coletta C., Le Trionnaire S., Perry A., Wood M.E., Whiteman M., Szabo C. (2014). AP39, a novel mitochondria-targeted hydrogen sulfide donor, stimulates cellular bioenergetics, exerts cytoprotective effects and protects against the loss of mitochondrial DNA integrity in oxidatively stressed endothelial cells in vitro. Nitric Oxide.

[13] Gerő D., Torregrossa R., Perry A., Waters A., Le-Trionnaire S., Whatmore J.L., Wood M., Whiteman M. (2016). The novel mitochondria-targeted hydrogen sulfide (H_2_S) donors AP123 and AP39 protect against hyperglycemic injury in microvascular endothelial cells in vitro. Pharmacol. Res..

[14] Karwi Q.G., Bornbaum J., Boengler K., Torregrossa R., Whiteman M., Wood M.E., Schulz R., Baxter G.F. (2017). AP39, a mitochondria-targeting hydrogen sulfide (H_2_S) donor, protects against myocardial reperfusion injury independently of salvage kinase signalling. Br. J. Pharmacol..

[15] Dugbartey G.J., Penney L.N., Mills L., Zhang M.Y., Juriasingani S., Major S., McLeod P., Liu W., Haig A., Wood M.E. (2025). AP39, a novel mitochondria-targeted hydrogen sulfide donor, promotes cutaneous wound healing in an in vivo murine model of acute frostbite injury. Biomed. Pharmacother..

[16] Ding Y., Ding X., Zhang H., Li S., Yang P., Tan Q. (2022). Relevance of NLRP3 Inflammasome-Related Pathways in the Pathology of Diabetic Wound Healing and Possible Therapeutic Targets. Oxidative Med. Cell. Longev..

[17] Wang X., Li X., Liu J., Tao Y., Wang T., Li L. (2024). Lactobacillus Plantarum Promotes Wound Healing by Inhibiting the NLRP3 Inflammasome and Pyroptosis Activation in Diabetic Foot Wounds. J. Inflamm. Res..

[18] Al Tahan M.A., Marwah M.K., Dhaliwal M., Diaz Sanchez L., Shokr H., Kaur M., Ahmad S., Badhan R.K.S., Dias I.H.K., Sanchez-Aranguren L. (2025). Novel AP39-Loaded Liposomes Sustain the Release of Hydrogen Sulphide, Enhance Blood-Brain Barrier Permeation, and Abrogate Oxidative Stress-Induced Mitochondrial Dysfunction in Brain Cells. Drug Des. Dev. Ther..

[19] Li L., Rose P., Moore P.K. (2011). Hydrogen sulfide and cell signaling. Annu. Rev. Pharmacol. Toxicol..

[20] Jacob S., Nair A.B., Shah J., Sreeharsha N., Gupta S., Shinu P. (2021). Emerging Role of Hydrogels in Drug Delivery Systems, Tissue Engineering and Wound Management. Pharmaceutics.

[21] Lin X., Zhang X., Wang Y., Chen W., Zhu Z., Wang S. (2025). Hydrogels and hydrogel-based drug delivery systems for promoting refractory wound healing: Applications and prospects. Int. J. Biol. Macromol..

[22] Kapanya A., Somsunan R., Molloy R., Jiranusornkul S., Jongpaiboonkit L., Kong Y., Baurecht D. (2019). Sodium 2-acrylamido-2-methylpropanesulfonate/gelatin hydrogels for use as wound dressings: Preparation, characterization and cytocompatibility. Biomed. Phys. Eng. Express.

[23] Zhang Q.Y., Yan Z.B., Meng Y.M., Hong X.Y., Shao G., Ma J.J., Cheng X.R., Liu J., Kang J., Fu C.Y. (2021). Antimicrobial peptides: Mechanism of action, activity and clinical potential. Mil. Med. Res..

[24] Hariharan K., Patel P., Mehta T. (2022). Surface modifications of gold nanoparticles: Stabilization and recent applications in cancer therapy. Pharm. Dev. Technol..

[25] Adnan S.B., Maarof M., Fauzi M.B., Md Fadilah N.I. (2025). Antimicrobial Peptides in Wound Healing and Skin Regeneration: Dual Roles in Immunity and Microbial Defense. Int. J. Mol. Sci..

[26] Rashid F., Carter P., Childs S. (2025). Overview of Hydrogels and the Use of Hyaluronic Acid-Based Hydrogels in Pharmaceutical Transdermal Delivery Systems and Topical Cosmetic Skin Applications. Cosmetics.

[27] Rungrod A., Kapanya A., Punyodom W., Molloy R., Meerak J., Somsunan R.J.B. (2021). Synthesis of poly (ε-caprolactone) diacrylate for micelle-cross-linked sodium AMPS hydrogel for use as controlled drug delivery wound dressing. Biomacromolecules.

[28] Nalampang K., Panjakha R., Molloy R., Tighe B.J. (2013). Structural effects in photopolymerized sodium AMPS hydrogels crosslinked with poly(ethylene glycol) diacrylate for use as burn dressings. J. Biomater. Sci. Polym. Ed..

[29] Marwah M.K., Hindalekar Y.S., Rana K., Shokr H., Al Tahan M.A., Sanchez-Aranguren L., Sarr M., Kainth R., Babaei P., Asif H. (2026). Naringenin Loaded Hydrogel Supports Wound Repair in a Cell Model of Diabetic Skin. Pharm. Res..

[30] Marwah M.K., Manhoosh B., Shokr H., Al Tahan M.A., Stewart R., Iqbal M., Sanchez L.D., Abdullah S., Ahmad S., Wang K. (2023). Transdermal delivery of mitochondrial-targeted hydrogen sulphide donor, AP39 protects against 6-hydroxydopamine-induced mitochondrial dysfunction. Eur. J. Pharm. Biopharm..

[31] Marwah M.K., Shehzad S., Shokr H., Sacharczuk J., Wang K., Ahmad S., Sanchez-Aranguren L. (2022). Novel controlled-release polylactic-co-glycolic acid (PLGA) nanoparticles for sodium thiosulphate, a hydrogen sulphide donor, retains pro-angiogenic potential of hydrogen sulphide. J. Exp. Nanosci..

[32] Marwah M.K., Shokr H., Rana K.S., Hindalekar Y.S., Kainth R., Babaei P., Ahmad S. (2025). Formulation and In Vitro Characterisation of Withaferin A-Loaded Liposomal Gels for the Topical Management of Chronic Inflammatory Skin Conditions. Br. J. Biomed. Sci..

[33] Sanchez-Aranguren L., Grubliauskiene M., Shokr H., Balakrishnan P., Wang K., Ahmad S., Marwah M.K. (2022). Sodium Thiosulphate-Loaded Liposomes Control Hydrogen Sulphide Release and Retain Its Biological Properties in Hypoxia-like Environment. Antioxidants.

[34] Marwah M.K., Balakrishnan P., Junaid S., Ahmad S., Shokr H., Upadhya M., Sanchez-Aranguren L. (2026). PLGA nanoparticle-integrated microneedles for controlled transdermal delivery of ADT-OH to ameliorate endothelial dysfunction in vitro. Int. J. Pharm..

[35] Zhao X., Liu L., An T., Xian M., Luckanagul J.A., Su Z., Lin Y., Wang Q. (2020). A hydrogen sulfide-releasing alginate dressing for effective wound healing. Acta Biomater..

[36] Marwah M.K., Shokr H., Sanchez-Aranguren L., Badhan R.K.S., Wang K., Ahmad S. (2022). Transdermal Delivery of a Hydrogen Sulphide Donor, ADT-OH Using Aqueous Gel Formulations for the Treatment of Impaired Vascular Function: An Ex Vivo Study. Pharm. Res..

[37] Gefen A., Weihs D., Fremau A., Eynde Y.V.D., Torfs E. (2025). Rheological Assessment for Determining Form Stability of Wound Dressings. Int. Wound J..

[38] Zhao X., Xu H., Sun Y., Yang Y., Guo B. (2025). Self-Adaptive Wound Dressings for Wound Healing and Repair. Adv. Mater..

[39] Dong D., Mao L., Qin Z., Guo Y., Yu J., Hu X., He J., Feng S., Zhang M., Liu Y. (2025). A simple and effective hydrogel dressing for advanced management of full-thickness skin wound by multi-functional strategies. J. Nanobiotechnol..

[40] Shen Z., Ma N., Xu J., Wang T. (2024). Metal-ion-controlled hydrogel dressing with enhanced adhesive and antibacterial properties for accelerated wound healing. Mater. Today Bio.

[41] Palierse E., Mihailescu A.M., Bergquist I., Persson C., Aramesh M. (2025). Tuning the mechanical properties and printability of viscoelastic skin-derived hydrogels for 3D cell culture. Biomater. Sci..

[42] Li M., Li C., Blackman B.R.K., Eduardo S. (2022). Mimicking nature to control bio-material surface wetting and adhesion. Int. Mater. Rev..

[43] Daengmankhong J., Ross S., Pinthong T., Mahasaranon S., Viyoch J., Tighe B.J., Derry M.J., Topham P.D., Ross G. (2024). Water-soluble macromers based on 2-acrylamido-2-methyl-1-propanesulfonic acid sodium salt (Na-AMPS) for rapid in situ hydrogel film formation. J. Polym. Chem..

[44] Higuchi T. (1960). Physical chemical analysis of percutaneous absorption process from creams and ointments. J. Soc. Cosmet. Chem..

[45] Huang G., Gao J., Hu Z., John J.V.S., Ponder B.C., Moro D. (2004). Controlled drug release from hydrogel nanoparticle networks. J. Control. Release.

[46] He H., Cao X., Lee L.J. (2004). Design of a novel hydrogel-based intelligent system for controlled drug release. J. Control. Release.

[47] Sanchez-Aranguren L., Hassanzadeh Moghadam B., Al Tahan M.A., Kruszyna K., Baxandall J., Shokr H., Marwah M.K. (2026). PLGA-Encapsulated Mitochondrial Hydrogen Sulphide Donor, AP39, Resolve Endothelial Inflammation via Mitochondria-Targeted Bioenergetic and Redox Modulation. Clin. Bioenerg..

[48] Balakrishnan P., Junaid S., Ahmad S., Wang K., Hindalekar Y.S., Shokr H., Upadhya M., Hopkins S., Sacharczuk J., Singh Rana K. (2025). Novel microneedle patches for transdermal delivery of AP39, a hydrogen sulphide donor, in the treatment of scenarios mimicking neurological disorders. J. Pharm. Investig..

[49] Sanchez-Aranguren L., Marwah M.K., Nadeem S. (2021). Neuroprotective effects of mitochondrial-targeted hydrogen sulphide donor, AP39 on H_2_O_2_-induced oxidative stress in human neuroblastoma SHSY5Y cell line. Adv. Redox Res..

[50] Parker A.M., Lees J., Phang J., Song S., Lim S.Y., De Blasio M.J., Ritchie R. (2024). Novel mitochondria-targeted therapy AP39 limits mitochondrial dysregulation and diastolic dysfunction in 2D & 3D human models of diabetic cardiomyopathy. Eur. Heart J..

[51] Teodoro J.S., Nunes S., Rolo A.P., Reis F., Palmeira C.M. (2019). Therapeutic options targeting oxidative stress, mitochondrial dysfunction and inflammation to hinder the progression of vascular complications of diabetes. J. Front. Physiol..

[52] Covarrubias A.E., Lecarpentier E., Lo A., Salahuddin S., Gray K.J., Karumanchi S.A., Zsengellér Z.K. (2019). AP39, a modulator of mitochondrial bioenergetics, reduces antiangiogenic response and oxidative stress in hypoxia-exposed trophoblasts: Relevance for preeclampsia pathogenesis. Am. J. Pathol..

[53] Paul B.D., Snyder S.H., Kashfi K. (2021). Effects of hydrogen sulfide on mitochondrial function and cellular bioenergetics. Redox Biol..

[54] Munteanu C., Turnea M.A., Rotariu M. (2023). Hydrogen Sulfide: An Emerging Regulator of Oxidative Stress and Cellular Homeostasis-A Comprehensive One-Year Review. Antioxidants.

[55] Xie Z.Z., Liu Y., Bian J.S. (2016). Hydrogen Sulfide and Cellular Redox Homeostasis. Oxidative Med. Cell. Longev..

[56] Lee E.G., Luckett-Chastain L.R., Calhoun K.N., Frempah B., Bastian A., Gallucci R.M. (2019). Interleukin 6 Function in the Skin and Isolated Keratinocytes Is Modulated by Hyperglycemia. J. Immunol. Res..

[57] Karaman Y., Kaya-Yasar Y., Eylem C.C., Onder S.C., Nemutlu E., Bozkurt T.E., Sahin-Erdemli I. (2023). The effect of mitochondria-targeted slow hydrogen sulfide releasing donor AP39-treatment on airway inflammation. Eur. J. Pharmacol..

[58] Pomierny B., Krzyżanowska W., Jurczyk J., Skórkowska A., Strach B., Szafarz M., Przejczowska-Pomierny K., Torregrossa R., Whiteman M., Marcinkowska M. (2021). The Slow-Releasing and Mitochondria-Targeted Hydrogen Sulfide (H_2_S) Delivery Molecule AP39 Induces Brain Tolerance to Ischemia. Int. J. Mol. Sci..

[59] Caley M.P., Martins V.L., O’Toole E.A. (2015). Metalloproteinases and Wound Healing. Adv. Wound Care.

[60] Chen J., Qin S., Liu S., Zhong K., Jing Y., Wu X., Peng F., Li D., Peng C. (2023). Targeting matrix metalloproteases in diabetic wound healing. Front. Immunol..

[61] Wolosowicz M., Prokopiuk S., Kaminski T.W. (2025). Matrix Metalloproteinase-9 (MMP-9) as a Therapeutic Target: Insights into Molecular Pathways and Clinical Applications. Pharmaceutics.

