# Supplementary material for: Mitochondria-Targeted Hydrogen Sulphide Delivery via an Adhesive Hydrogel Modulates Inflammation and Oxidative Stress in Diabetic Wounds
Source: Gels. 2026 Mar 17;12(3):251. doi: 10.3390/gels12030251 (PMC13025655; doi:10.3390/gels12030251)
Supplement: Supplementary file 1 [file gels-12-00251-s001.zip › gels-4179435-supplementary.pdf]

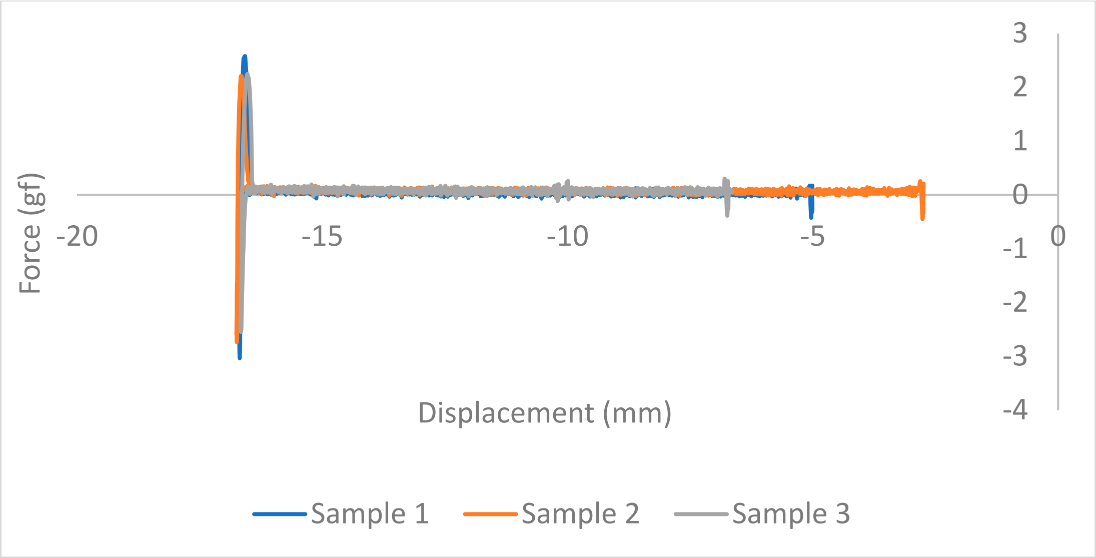

Supplementary Figure S1. A single representative force-displacement curve for an adhesive hydrogel loaded with AP39. Each curve illustrates the measurement from one sample and one time.

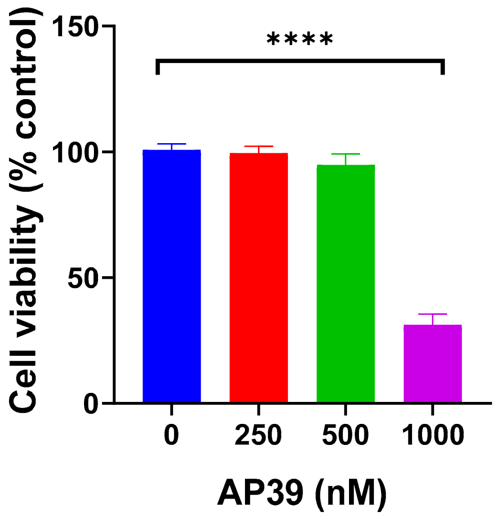

Supplementary Figure S2: Cell viability of HaCats following 24-hour exposure to hydrogel permeate containing varying concentrations of AP39. Cell viability was assessed following trypan blue staining and cell counting. Data represent mean ± SD (n = 6). Statistical significance: ****$p < 0.0001$.
